# A Framework to Simplify Combined Sampling Strategies in Rosetta

**DOI:** 10.1371/journal.pone.0138220

**Published:** 2015-09-18

**Authors:** Justin R. Porter, Brian D. Weitzner, Oliver F. Lange

**Affiliations:** 1 School of Medicine, Washington University in St. Louis, Missouri, Washington, United States of America; 2 Department of Chemical and Biomolecular Engineering, Johns Hopkins University, Baltimore, Maryland, United States of America; 3 Lehrstuhl für Biomolekulare NMR-Spektroskopie, Fakultät Chemie, Techniche Universität München, Munich, Germany; UMR-S665, INSERM, Université Paris Diderot, INTS, FRANCE

## Abstract

A core task in computational structural biology is the search of conformational space for low energy configurations of a biological macromolecule. Because conformational space has a very high dimensionality, the most successful search methods integrate some form of prior knowledge into a general sampling algorithm to reduce the effective dimensionality. However, integrating multiple types of constraints can be challenging. To streamline the incorporation of diverse constraints, we developed the Broker: an extension of the Rosetta macromolecular modeling suite that can express a wide range of protocols using constraints by combining small, independent modules, each of which implements a different set of constraints. We demonstrate expressiveness of the Broker through several code vignettes. The framework enables rapid protocol development in both biomolecular design and structural modeling tasks and thus is an important step towards exposing the rich functionality of Rosetta’s core libraries to a growing community of users addressing a diverse set of tasks in computational biology.

## Introduction

The core task in modeling structures of biological macromolecules—including structure prediction[1,2], structure determination from sparse experimental data[3,4], and biomolecular design[5–7]—is searching conformational space for low-energy configurations. However, the number of dimensions grows with the size of the atomic system, making the conformational space of typical systems impossible to sample exhaustively. As a result, successful sampling algorithms incorporate as much information as possible about the biomolecular system in question to focus the search on the most productive regions of conformational space.

Prior knowledge is highly diverse, both in origin and in its implications for sampling, and numerous successful methods in macromolecular modeling are based on incorporating one or several different types of prior knowledge into the model. Some methods, for instance, rely on atomic coordinates from experimental data (*e*.*g*. I-TASSER[8], RosettaCM[9], numerous design strategies [7,10,11]). Even when no homologous structures are available, some unrelated experimental structures still contain small regions of structural similarity, which provide a set of reasonable backbone torsional angles for that sequence (“fragment insertion” [12] [13]). A different strategy, used by systems like the Integrated Modeling Platform (IMP)[14], is to build scoring potentials to bias in favor of agreement with experimental data. Even Molecular Dynamics (MD) [1,15] simulations of proteins, which emphasize reliance on physics-based potentials, usually fix bond lengths and angles[16]. All of the above strategies combine some general-purpose sampling algorithm (*e*.*g*. MD, Metropolis Monte Marlo) with prior knowledge to prioritize the most promising regions of conformational space.

To be useful, prior knowledge must typically be integrated with a general sampling protocol, and this process is not always straightforward. Several integration strategies exist. A naïve approach might be to filter general algorithm output *ex post facto*, rejecting all output from the general algorithm that conflicts with prior knowledge. As the number of independent filtering conditions grows, however, the fraction of output that passes all filters quickly approaches zero, rendering this approach impractical in most situations.

Correcting structures that deviate from the conditions proscribed by prior knowledge during sampling is more scalable than the filtering of entire trajectories. One implementation of this strategy is the restraint: unacceptable states are assigned a score penalty, biasing the conformation away from those states. This strategy, while an improvement on filtering, also scales poorly. In cases where restraint targets are far from the starting configuration, the additional score penalty may frustrate sampling. That said, this approach is viable in many cases, and has been used to great effect in state-of-the-art prediction[4,17,18] and design[6,7,11,19] simulations in Rosetta, as well as in other software suites like HADDOCK[17] and IMP[14].

A more scalable strategy is to simply avoid generating unacceptable states in the first place. If sampling can be modified to produce only acceptable states, additional constraints require no additional work. In Rosetta, this strategy frequently takes the form of a change of coordinates. To maintain ideal bond lengths and angles, Rosetta represents the protein in internal coordinates—the positions of the atoms are represented as bond lengths, angles, and torsions, rather than in Cartesian coordinates. As a result, a sampling protocol can passively maintain fixed bond lengths and angles by restricting changes to torsional angles. In contrast, systems using a Cartesian space representation must do significant work to achieve the same result. [20,21] The internal coordinates approach is highly scalable, as adherence to each additional constraint comes at no additional cost.

While they scale well, these systems using implicit constraints place heavy demands on the protocol designer. First, the protocol designer must conceive of an appropriate coordinate system. Second, the protocol must convert from this coordinate system into Cartesian coordinates, because long-range pairwise inter-atomic interactions are evaluated in Cartesian space. For example, consider a perturbation to a backbone torsional angle in the second residue of a protein. In internal coordinates, the change is unambiguous. In Cartesian coordinates, however, it is ambiguous whether this change affects the Cartesian position of all atoms N-terminal to this torsional angle, or all atoms C-terminal. This requires a choice for the direction of propagation for internal coordinate changes in Cartesian space. This choice of direction is referred to as the “folding direction”.

Counterintuitively, the folding direction does not always need to follow the path of bonds. To allow for the direct sampling of non-bonded spatial relationships, Bradley *et al*.[22] introduced the *jump*. A jump separates two arbitrary segments of peptide and sets the spatial relationship between the two segments directly. Instead of always folding from N- to C-terminus along the peptide chain, the propagation of Cartesian coordinates can arbitrarily “jump” to distant regions of sequence space. One of the many applications of the jump in Rosetta is the direct control of β-strand pairings. A jump is placed between two paired residues that directly relates their respective peptide bonds, effectively “short-circuiting” the intervening residues in sequence space. The paired atoms are distant in sequence space, but adjacent in the path of folding. The relative Cartesian positions of jump-paired atoms are unaffected by changes in torsional degrees of freedom (DoF). Rosetta uses the *fold tree* to represent this choice of peptide segments and jumps.

In Rosetta3[23], the core representation of molecules includes the fold tree, which is a directed, rooted, acyclic graph. The direction of an edge tracks the folding direction, and the root determines the position of the entire system relative to the origin. A key property of fold trees is the absence of any cycles, which prevents the existence of two valid coordinate propagation paths that lead to the same atom. As a result, when a jump is introduced between two atoms that are connected by any number of chemical bonds, that chain of bonds must be broken. This is known as a *cut* and, typically, the cuts must be *repaired* at the end of a trajectory for the result to have physical significance. Thus, introducing jumps comes at a cost: the jumped position is now ‘fixed,’ but the ideal geometry of the peptide chain around the cutpoint is lost. This geometry must then be constrained by filtering, biasing potentials, or an *ex post facto* correction.

Clever use of the fold tree’s flexibility and expressiveness has been the technical underpinning for many of Rosetta’s diverse successes. One or more sampling strategies, each implemented by a “Mover,” are applied to the appropriate DoFs modeled by the fold tree. Loop modeling strategies[24,25], for example, use a jump to bypass a loop, maintaining the geometry of the protein’s core while the Mover acts only to perturb surface loops. The loop is cut somewhere in the middle. RASREC[18], uses a similar fold tree layout but applies Movers that implement torsional fragment insertion *in lieu* of a loop closure scheme. As sampling converges on a core topology, RASREC holds fixed increasingly large portions of the protein core to save sampling time. Jumps can also be used to model protein–protein[26] and protein–ligand[27] interactions by controlling the rigid body degrees of freedom between two chemically unbound molecules. The dimensionality of symmetric assemblies has been greatly reduced by the judicious application of fold trees that allow for changes to easily be copied between symmetry partners [28], and inverse-rotamer fold tree construction has been of great benefit to enzyme design[19].

A factor that, while not encoded in the fold tree, drastically alters sampling is the extent to which degrees of freedom are moved. In a Rosetta simulation, degrees of freedom usually take the form of peptide chain torsion angles or jumps. Consider a large segment of protein structure that is constituted by disparate regions of sequence-space and must be held fixed—a search-space reducing strategy used by some successful design[19,29,30] and prediction[18,24,25,31] protocols. Not only must the discontinuous segments be “pinned” to one another using jumps, but the torsional degrees of freedom inside individual peptide segments must be constrained, too. In Rosetta, no system for communicating choices between Movers has emerged, and no mechanism exists to enforce such choices. Historically, these challenges have been managed at the individual protocol level; protocol designers must either implement their own custom-purpose enforcement schemes[28] or, much more commonly, carefully verify that their choices for DoF flexibility are correctly communicated to and honored by the algorithms that implement individual components of their sampling protocol.

Fold tree construction is challenging because it must integrate knowledge of the entire protocol. Both the fold tree layout and DoF flexibility depend on all constraints and, as a result, cannot be defined in a pairwise-independent manner as score penalties or filters can. For this reason, many protocol designers opt to simply sketch out the correct fold tree by hand and hard-code it into the protocol. Of course, requiring the developer to hard-code an appropriate fold tree does not scale well for large numbers of targets with different residue numbers, makes it difficult to make choices that vary between trajectories (*e*.*g*. a cut placed in a randomized location). For example, it is not even possible to implement some successful protocols with hard-coded fold trees, including the algorithms described in refs. [22] and [32], which use randomized fold trees to sample the variety of folding directions that are consistent with a particular constraint set on a particular target.

The most common strategy for constructing a fold tree is to write a custom fold tree setup routine. These routines examine the target of interest and, knowing the precise number and nature of the active constraints, lay out a compatible fold tree. Although this strategy scales well for multiple targets of the same type, it does not scale well when additional constraints are added because an entirely new routine must be developed. For some particularly useful or common cases, a number of “remix protocols” have been developed. Notable examples include: Fold and Dock[33], which combines symmetric docking with *ab initio* folding; SnugDock[31], which combines loop modeling with multibody docking; and RosettaRemodel[30], which seeks to unify a large number of design-relevant sampling algorithms under one interface. As the number of available constraint types and basic sampling strategies available in Rosetta grows, the combinatorial explosion of possibilities renders it unreasonable to develop and maintain custom fold tree protocols using the remix strategy.

To avoid the development work involved with the remix strategy, developers resort to chaining sampling modules together sequentially. Using this strategy, each sampling module independently calculates and manages its own fold tree and DoF accessibility. Then, after its sampling move is complete, it resets the fold tree to its original state. We refer to this as the “set-sample-reset” pattern, and it is currently the most commonly used strategy in RosettaScripts[34]. This strategy has low developer time investment, but it can quickly become problematic as the number of sampling strategies in concurrent use grows. Each strategy owns its own fold tree, which encodes its own constraints, but only one fold tree can be active at any given time: the one that was just “set” for the sampling strategy that is going to be used next. Consequently, it is impossible to enforce the constraints for an inactive fold tree. Unless the two constraints are compatible by happenstance—raising the question as to why two constraints were required in the first place—the system finds itself in the Sisyphean situation of correcting one constraint violation only to violate the other.

In this paper, we present the Broker: a tool that generates a “consensus fold tree,” a fold tree that satisfies the needs of all constraints without the need for additional application code development. We demonstrate the use of our tool through several examples, which are focused on underscoring the flexibility and expressiveness of the system, rather than being the solution to outstanding scientific problems. The Broker provides an API—accessible in Rosetta C++, RosettaScripts XML[34], and PyRosetta[35]—that allows sampling algorithms to describe requirements of the fold tree and the extent of control they require over the DoFs implied by that fold tree. Once all specifications are made, the Broker produces a fold tree that satisfies all constraints requested by its client Movers. The framework does not require that clients have knowledge about one another—instead they express only their own requirements as a formalized claim. As a result, clients are modular and can be added and removed without writing new code. This reduces “remix protocol” development to a scripting task, and enables rapid prototyping of combined sampling strategies in a way heretofore impossible in Rosetta.

## Results

In the following three sections we demonstrate the expressiveness of the Broker system through the discussion of several short code vignettes. Then, in the final section, we describe the design and implementation of the Broker system itself.

### *Ab initio* with Multiscale Constraints

In this vignette, we demonstrate the combination of two types of constraints, that represent information on multiple scales. On the atomic scale, we use chemical shift fragments, 3–9 residue long segments of polypeptide chain harvested from existing crystallographic structures and selected by matching their predicted chemical shifts with experimental NMR data[36]. We also fix a small region using experimental crystallographic coordinates. On a more coarse-grained scale, we constrain the β-strand topology of the protein. The later stages of RASREC[18], for example, use β-strand pair constraints to intensify sampling of topologies deemed successful in earlier stages. This strategy has proven essential for extending structure determination with CS-Rosetta to larger protein structures[32,37]. These multi-scale constraints act together in a single, simultaneous *ab initio*-style folding of ubiquitin.

**Fig 1:**
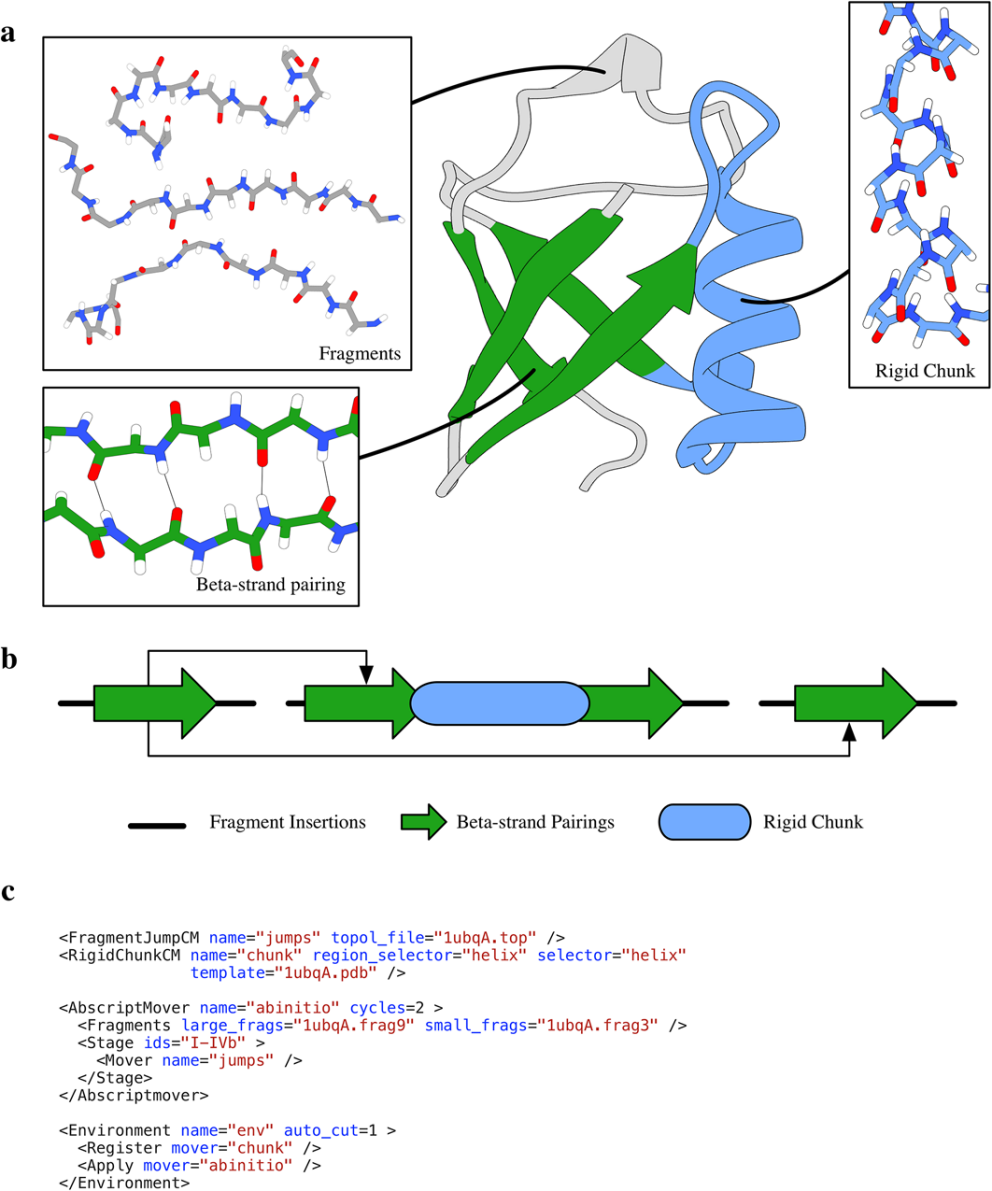
Multi-resolution constraints in a simple folding protocol. a) The crystal structure of ubiquitin is color- coded and annotated by the sampling procedures applied to each region. Green strands indicate regions of β-strand pair sampling, the blue region has fixed internal coordinates drawn from the native crystal structure, and grey and green are subject to fragment insertion using fragments from chemical shifts. b) One possible fold tree generated by the Broker to satisfy the constraints given in (a). Black pointing arrows represent jumps between β strands, which are represented by green block arrows. Breaks in the underlying black line indicate possible chain break locations. The fixed region is indicated by the blue rounded rectangle. c) The main part of a RosettaScripts XML script that implements this protocol. The full script is available in the supplement and the script along with all required files is available in the protocol capture.

Using the Broker system, information is introduced modularly as constraints, each set of which is represented by a different client Mover. Fig 1A shows this protocol as color-coded regions on a crystal structure of ubiquitin. We chose ubiquitin here for its simplicity and pedagogical value. In fact, ubiquitin is small enough that the lowest energy structures generated with the CS-Rosetta abrelax protocol published in ref [36] have sub-Ångström backbone RMSDs.

β-strand constraints are introduced using a Broker-compatible ClientMover (FragmentJumpCM) that takes information about one or more possible β-strand pairings and, using the Broker system, introduces a jump that “pins” the peptide bond planes of the paired residues to one another in the rigid body geometry of known β-strand pairings. Because of the mathematical properties of the fold tree implementing the “pin,” only two residues can be pinned per strand-pair (rather than all residues predicted to be involved). Making a different choice of pairs in each trajectory, however, allows sampling of the entire space of possible pairs within the ensemble of generated structures. Then, when the FragmentJumpCM is invoked to produce a Monte Carlo trial move (typically 10^3^−10^4^ times per trajectory), it samples jump geometries from a database of transforms observed in crystal structures. This approach, introduced in [22], has proven useful in the past decade[4,18] and has seen wide adoption. In the protocol capture for this paper (found at Rosetta/demos/protocol_capture/broker/, were “Rosetta” is the Rosetta bundle found at https://www.rosettacommons.org/software/license-and-download), we include a Python script that generates topology files using the format introduced in [18].

For purely demonstrative purposes, we add an additional set of constraints that fix the central helix and adjacent loops (1ubq[38] residues 17–40) of ubiquitin to the native conformation found in the crystal structure. In a real-world application, this structural information could come from a homologous structure. We include the constraints associated with this information using a *rigid chunk* (RigidChunkCM). This client Mover uses the Broker to claim exclusive access to the torsion DoFs in this region, and then sets all such DoFs to match those in a *template* (in this example, the crystal structure 1ubq). Because the Broker grants the Mover exclusive access to the DoFs in question, the Mover can “set and forget” them, being guaranteed that they will receive no further sampling. As a result, no further time is spent sampling this region.

The resultant fold tree scheme is shown in Fig 1B. The FragmentJumpCM places a jump between two paired residues for each β-strand pair. The RigidChunkCM fixes the torsional angles in the helix and adjacent loops to the template values and prevents cuts in this region. In this example, the Broker itself is responsible for making all the cuts, which are randomized on a per-model basis.

The relevance of this example is not the modeling trajectories themselves, but rather that it demonstrates how degrees of freedom can be constrained in a plug-and-play fashion without the need for additional development. This modularity is apparent in the structure of the XML that implements this protocol (Fig 1C). Removing any group of constraints is as simple as removing the respective Mover from the XML script. Similarly, including additional constraints is as uncomplicated as declaring a new Mover and adding it to the BrokeredEnvironment block in the XML script.

### Domain-Insertion Modeling

**Fig 2:**
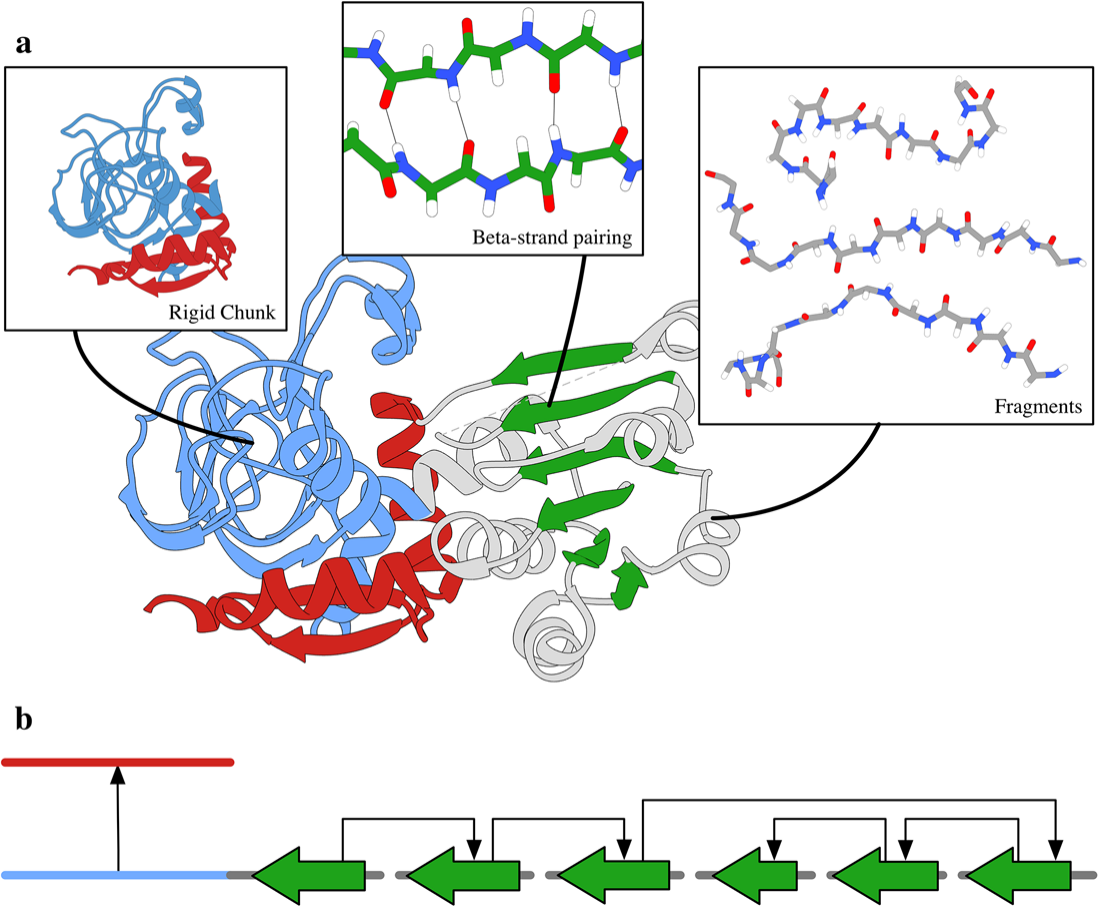
The use of fixed chunk regions and fragment jumps for domain insertion modeling. a) The structure of the domain insertion protein YahK (PDB code 1uuf) color-coded to indicate the regions that various client Movers act on. Gray indicates traditional torsion fragment insertion and loop closure, and green indicates torsion and β-strand fragment insertion. Blue and red are, respectively, the N- and C-terminal portions of the host domain. Those regions are fixed. b) A possible consensus fold tree (note that many fold trees are valid consensus fold trees depending upon the choices made at run time for cut placement, β-strand pairing choices, *etc*.). The two discontinuous chains of the host domain (color-coded as above) are fixed in their relative geometry by the jump that connects the C- and N-terminal regions directly. The insert is broken into multiple stretches (grey and green) by the jumps created for β-strand pairing (green).

Because domain insertion proteins occur frequently in nature[39], modeling them has been a subject of considerable interest in both the Rosetta community[40,41] and the structural biology community at large[42,43]. In this vignette, we demonstrate the use of rigid body and jump fragment constraints to fold an inserted domain of unknown structure onto a host domain with a known structure. The protein used in this example is an *E*. *coli* zinc-type alcohol dehydrogenase-like protein (PDB code 1uuf) which consists of a Rossman-folded domain inserted into a loop of a GroES-like domain (Fig 2A).

Underscoring the flexibility of our tools, this vignette uses the same client Movers to implement the constraints as the previous example to implement a protocol that would have required custom development without the Broker (*e*.*g*. ref. [41]). Unlike the previous vignette, where the rigid region of the protein was contiguous in sequence space, the host domain in this example is not: it has two discontinuous segments of peptide (N- and C-terminal regions) that must be kept fixed relative to each other. To hold this discontinuous chunk fixed, both segments are fixed internally as before, and then connected by a fixed jump. Thus, by rewiring the folding direction to bypass the inserted domain, no changes in the inserted domain residues can modify the rigid body relationship between the two segments. The flexible inserted domain is then folded *in situ* using torsion and β-strand fragment insertion. Fig 2A provides a visual overview of this protocol.

The fold tree for this vignette includes a jump between the N- and C-terminal segments of the host domain and numerous jumps that enforce the native β-sheet topology of the Rossman fold of the inserted domain. Furthermore, the cuts are placed only in the inserted domain by request of the rigid chunk client Mover but, as before, the Broker manages cut placement automatically. Fig 2B shows a schematic of one possible fold tree for this vignette; several fold trees are possible depending upon which β-strand connections are chosen at runtime to be modeled explicitly.

It is a mere scripting exercise to add additional fixed regions from this template or additional chunks from other templates. Such additions could produce behavior similar to the first stage of RosettaCM[9] or to a single iteration of I-TASSER[8], both of which build up a structure prediction using many small, experimentally-solved protein chunks in a sort of “chunk assembly” algorithm.

### Flexible Backbone Multibody Docking

In this final vignette, we use the Broker to assemble a flexible-backbone docking protocol. Inspired by SnugDock[31], an algorithm for high-resolution refinement of antibodies, we implement a multi-body, flexible-backbone refinement protocol uses prior knowledge about antibody structural biology. Specifically, SnugDock treats all CDR loops differently and differentiates between antibody-antigen docking and heavy chain-light chain docking. Most of these behaviors are reproduced in our vignette’s SnugDock-inspired algorithm.

**Fig 3:**
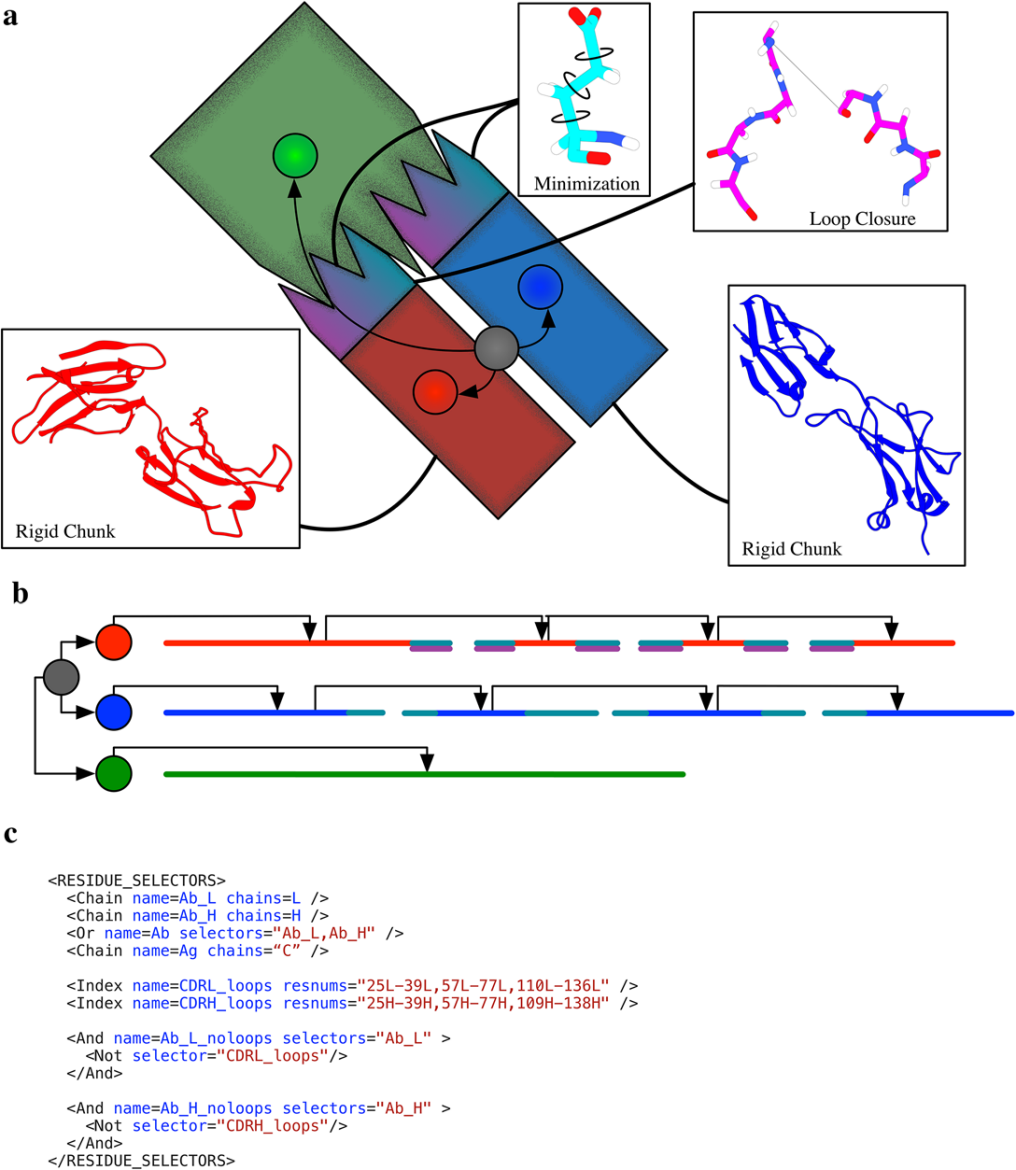
A SnugDock-inspired antibody modeling protocol configuration. a) An antibody heavy (red) and light (blue) chain in complex with an antigen (green), which interacts with the antibody’s CDR loops (cyan). Call-outs identify the sampling procedures that are active on this structure using this protocol, and colors indicate the regions that each prodcedure targets: the gradient-based minimizer (cyan), loop closure (magenta), and fixed backbone docking (red, green, and blue) of antibody chains and antigen. Additionally, the explicitly-monitored centers of mass of each of the three polypeptide chains are indicated (blue, green, and red circles) and each is docked to a central reference point (grey circle). b) The fold tree that underlies the situation in (a). Each chain is docked via its center of mass virtual residue (red, blue, and green circles) to a central virtual residue (grey circle). The antigen and antibody regions outside of CDR loops are fixed, whereas the CDR loops, each of which is interrupted by a cut, are flexibly modeled by minimization and subjected to loop closure. The color of the line indicates where Mover active: red, green, and blue are docking Movers, magenta and cyan are loop closure and minimization, respectively, and grey is unmoved. c) The definition of the ResidueSelectors used in the body of the script XML script. Note that many residue selectors are created using Boolean logic operators depending on other ResidueSelectors, making alterations straightforward.

Our protocol contains the following elements: sampling of the rigid-body orientations between all three interaction partners in the antibody-antigen complex (*i*.*e*. antibody heavy chain, antibody light chain, and antigen), loop refinement on only the CDRH loops[25,44], and minimization of all CDR loops. The antigen and antibody outside of the CDR loops are modeled with fixed backbones, because no client Mover is assigned to sample their DoFs. Additionally, we demonstrate the use of a novel center-of-mass (CoM) tracking system that, unlike previous implementations[33], does not require reconstruction of the fold tree as backbone atoms move. This protocol is represented schematically in Fig 3A.

In addition to the polypeptide chains of the three interaction partners, this example includes several virtual residues, seen best in the fold tree schematic in Fig 3B. A root virtual residue forms the center of a star pattern to which each of the three chains’ centers of mass is docked. Each CoM, also represented by a virtual residue, is then connected by a jump to the chain it tracks. During sampling, the CoM tracking Mover updates its virtual residue at the appropriate chain’s CoM without altering the location of any real atoms in space by changing both the incoming and outgoing jump(s) to the virtual residue. The Broker then associates docking client Movers with the jump between the root virtual residue and the center of mass virtual residue of the appropriate polypeptide chain.

This vignette also makes special use of a new, unpublished RosettaScript feature introduced by Andrew Leaver-Fay, called ResidueSelectors (Fig 3C), to determine the identity of the various chains and loops in the input structure and to give them human readable labels. (ResidueSelectors are used for their conciseness and clarity in all three vignettes, but this is the only vignette that actually requires them.) In this vignette, we chose chain identifiers ‘C’ for the antigen and ‘H’ and ‘L’ for the heavy and light chains of the antibody, respectively. These identifiers reflect the chain IDs in the input PDB file that is used to load the antibody structure. By using references to the chain labels, input becomes insensitive to the order of chains in the PDB file. Using the Aho residue numbering scheme[45], ResidueSelectors are also used to determine (at run time) which residues constitute which CDR loops. Thus, the script used to implement this vignette can be used on an arbitrary antibody PDB file using Aho numbering by adjusting only 1) file names and 2) chain identifiers, allowing for straightforward automation, which is indispensible for applications like web servers or extended benchmarking. Furthermore, this protocol could be trivially adapted to work with any numbering scheme by adjusting the residue selectors.

The algorithmic complexity of this vignette is typical of custom-built Rosetta applications. Lacking Broker support, the Rosetta 3 implementation of the SnugDock protocol[46] requires nearly 900 lines of C++ code at the protocol level (this includes only the definition of top-level Movers, the processing of input, and the handling of options) and a week of one author’s (BDW’s) time. In marked contrast, the protocol we describe is less than 100 lines of RosettaScript XML and was written in an afternoon. Furthermore, none of the code outside the XML script is specific to this vignette in any way—rather, all parts are general and interchangeable. It is thus a scripting exercise to adapt this protocol to another system or to test elaborations or wholesale replacements of any part of this protocol with another Broker-compatible sampling procedure.

### Design of the Broker

In this section, we discuss the design of the Broker and the motivation for the choices made in that design. We had three main design goals. The first was modularity. Components of a sampling protocol should be plug-and-play. Removal of one module should have the most limited possible effect on other modules. Similarly, adding additional behavior should be, to the greatest extent possible, as simple as adding the appropriate module. Such modularity has contributed to the success of knowledge-incorporating modeling platforms like IMP[14], and we sought to emulate it here.

The second goal was security. The result of broking represents a contract between all the client Movers, detailing their rights (*e*.*g*. DoFs to which it has exclusive access) and their responsibilities (*e*.*g*. accepting Broker’s fold tree layout). Guaranteeing the sanctity of the contract is indispensible. If one client were to change the fold tree, for example, all other clients would no longer be guaranteed that their DoF is present in the system. Similarly, some Movers require that a particular DoF remain fixed, which is meaningless if no system for enforcing that choice exists. Because of its enforcement of DoF accessibility, the broking system can provide functionality that is not possible under more permissive systems.

Our third goal was protocol transparency. It should be possible to get the “broad strokes” of a protocol in one place, and it should be possible to specify protocol-level details in a reasonably human-readable manner. To the extent possible, protocol designers should be able to express sets of residues in the terms they would use to describe those residues in natural language (*i*.*e*. chain letters rather than residue numbers), or using labels. As a result, designers can more easily specify their needs in a way that allows Rosetta to “just do the right thing.”

With these goals in mind, we chose to create a “broking layer” that sits between the Movers that implement the protocol and the resources that represent the protein (or macromolecule in question). *Client Movers*—the usual encapsulation of a sampling protocol in Rosetta—are registered with the *Broker*, which is responsible for producing the consensus fold tree and DoF accessibility based upon the *claims* provided by each client Mover. After the consensus fold tree and DoF accessibility are calculated by the Broker, each client Mover is given a *passport*, which contains information about which DoFs it has access to. The standard conformation—which stores the fold tree and internal coordinates for all atoms—is replaced with a *protected conformation*, which requires Movers to present a valid passport before modifying DoFs.

#### The Broking Process

**Fig 4:**
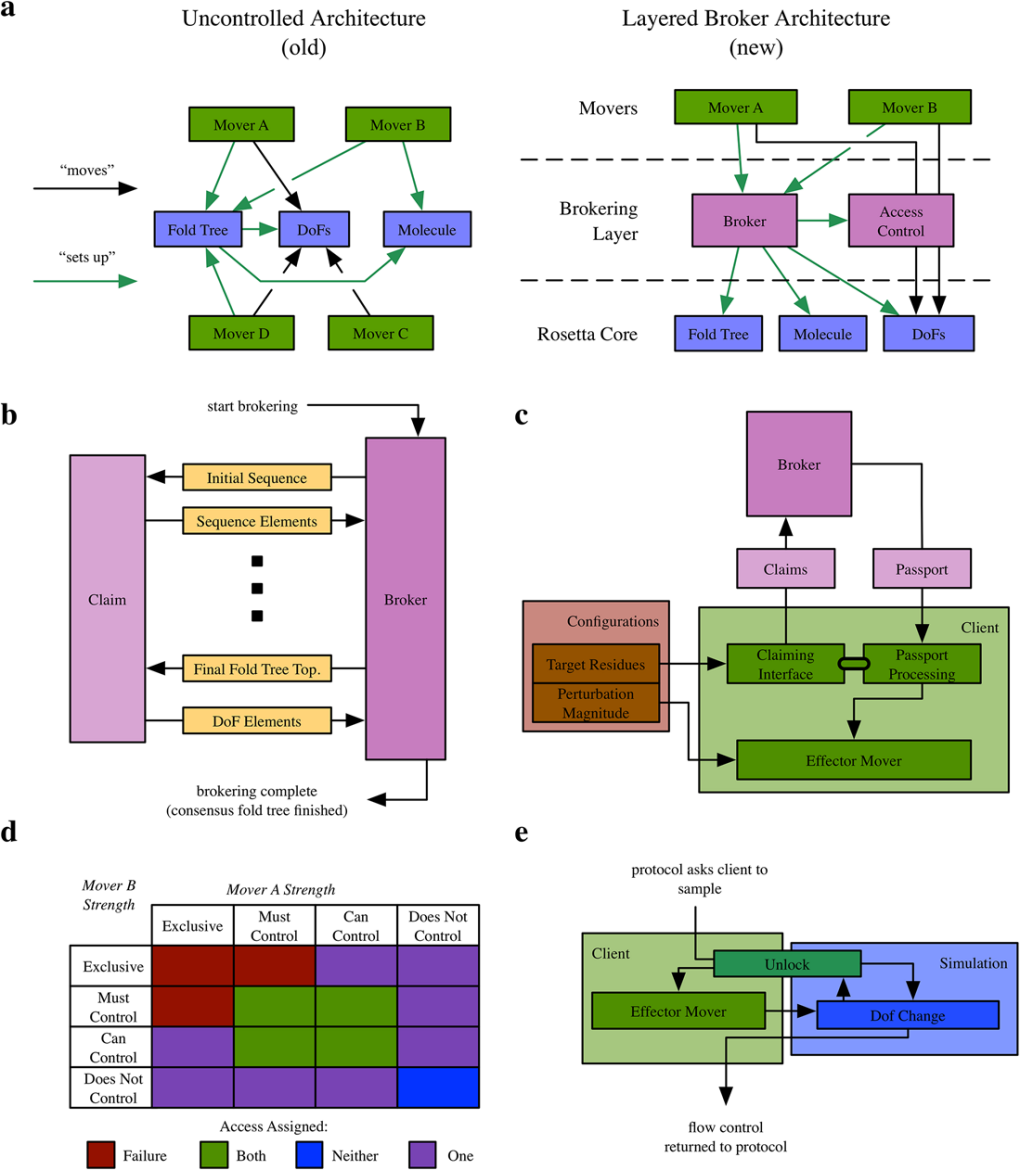
The design of the broking mechanism. a) right: the central resources of a Rosetta protocol (blue) are acted upon by many independent Movers (green) in an uncontrolled fashion. Movers differ in the actions they perform on these resources, including configuration (green arrows) and sampling (black arrows). Left: A Broker layer (purple) receives requests from numerous clients (green) using a standard interface, and configures the core resources appropriately. Access is restricted to these resources using an access control framework, but requests invisibly “pass through” this layer to avoid interface differences. b) The Broker communicates with client Movers by receiving claims and responding with a passport. c) Client Movers (light green) convert user-specified configurations (brown) into convert developer-friendly claims (light purple, left) through the claiming interface. The Broker (dark purple) converts claims into specific, machine-readable needs, which are processed and returned to the client Mover as a DoF passport (light purple, right). d) The DoF access assignment behavior of the Broker when two clients request access to the same DoF. If one Mover claims exclusive and another claims must control or exclusive, broking fails, because it is not possible to satisfy both. If one claims exclusive and the other claims can control, only the Mover claiming exclusive receives access. If a Mover claims “does not control,” it never receives access. In all other cases, both Movers receive access. e) The procedure by which the conformation validates a modification to a DoF. The client Mover creates an unlock, which is shared by the conformation and the Mover. Then, whenever to the conformation change the DoF, the conformation checks latest active unlock to ensure the active Mover has access to the changing degrees of freedom.

The result of the broking process is a contract among all client Movers about how to represent the DoFs and which Mover may modify which DoFs. Consequently, each client Mover must contribute information about its needs and accept communications regarding the brokered results. The Broker collects requirements from each client, constructs the consensus fold tree, and communicates results back to the clients (Fig 4A).

The broking process occurs in four distinct steps. First, the protocol registers each client. The Broker can accommodate any number of clients, in any order, at any time until protocol calls on the Broker to construct the consensus fold tree. Next, at “broking time,” each registered Mover is given the opportunity to assert any number of claims that describe its requirements for the fold tree through its “yield_claims” method. A client typically produces only a few claims, and they are usually straightforward to describe (a jump claim, for example, indicates the need for a jump). The Broker then integrates claims from all clients, constructs the consensus fold tree, and finally returns an individualized passport to the client. In the third step, the client interprets the data in the passport and configures itself to sample appropriately based on the final, consensus fold tree. For example, a docking Mover, which typically makes only one jump claim, must determine which of the jumps in the final fold tree belongs to it. Finally, the protocol invokes the client’s sampling procedure (the Mover’s “apply” function). Thus, he Broker does not have any control over when, how often, in which order, or even if clients are invoked. Order of invocation is determined at the protocol level after brokering, most commonly using a scripting interface like those available through PyRosetta[35] or RosettaScripts[34].

During the part of the broking process that is invisible to the clients (*i*.*e*. the construction of the consensus fold tree), each claim is converted into machine-readable *elements*, which are used to determine the placement of “virtual residues,” jumps, cuts, and the accessibility of each DoF to each Mover. The conversion occurs in distinct phases (Fig 4B), because certain fold tree properties rely on preceding properties (for example, for a jump that links two residues, those residues must first exist). Conceptually, each phase consists of the collection of some class of elements. In order, they are: sequence changes (addition/subtraction of residues), fold tree topology elements, DoF accessibility. In each phase of the element-production process, the Broker provides the claim with information about the status of broking, which the claim uses to inform the details of the elements it produces (*e*.*g*. the exact atom number of the DoF to claim). After collecting the elements created by each claim in each phase, the Broker integrates them into the nascent fold tree and returns information about how those elements were brokered to the claims, which begin the process anew for the next class of elements.

#### Clients and the Claiming API

A typical client Mover consists of two parts: a layer that interacts with the broking machinery, and an effector layer that contains the implementation of the client’s sampling behavior (Fig 4C). These parts can be integrated to a varying extent, depending on the needs of the individual client—many client Movers are little more than wrappers for existing Movers in Rosetta. As a consequence, it is a straightforward programming exercise to make an existing Mover Broker-compatible by modifying it to be a ClientMover or by producing an additional ClientMover that wraps it. A listing of available ClientMovers that could be used as examples can be found on the RosettaCommons wiki (https://www.rosettacommons.org/docs).

Once the client is configured by the protocol designer—via PyRosetta[35] or RosettaScripts[34], or through a C++ protocol—the client channels the input either to the effector, in the case of sampling configurations (*e*.*g*. a perturbation magnitude), or to claims, in the case of fold tree information (*e*.*g*. where a jump should be placed).

The way in which the degree of control over DoFs is expressed is particularly important. Every element that claims access to a DoF for a client Mover is associated with a *control strength*. Valid control strengths are *exclusive*, *must control*, *can control*, and *does not control*. When two clients claim the same DoF, the Broker’s behavior depends on the two claims’ control strengths (Fig 4D). This is of critical importance for the Broker system, because it allows Movers to communicate about their needs relative to other Movers without needing to know the identity of other Movers. Although control strength is a design choice to be made on a client Mover-specific basis, there are a few general principles for its use. In particular, the distinction between *exclusive* and *must* bears clarification. Both will fail if the client cannot be granted access to the specified DoF. The difference is that exclusive control strength requires that the client not only must get access to the DoF, but that no other client can be granted access to the DoF. For example, the rigid chunk client Mover requires that it be the only Mover to sample the internal DoFs of a particular region to perform its function (complete constraint of a region), and so it issues claims with exclusive rather than must control strength. In contrast, a Mover like the FragmentJumpCM must sample the beta strand pairings it creates, but it has no reason to prevent other Movers from doing the same. If two Movers produce conflicting control strengths over a particular DoF, the Broker recognizes this fact and produces an error, terminating the protocol and explaining which two Movers conflict at which DoF so that the user can easily debug the protocol.

Claiming information is represented in two ways: client Movers produce claims, and claims produce atomic claims called *elements*. This two-tiered structure enables the Broker to process elements on very granular level (each of the six degrees of freedom in a jump, for example, is brokered separately) while allowing clients to specify their needs in a broader, more human-readable manner. For instance, the jump claim actually generates elements for six degrees of freedom, one jump and one cut. Because this jump-claiming behavior is common to many clients, our design allows this code to be stored in the claim and shared among many client Movers. Furthermore, as researchers develop new types of behaviors that are not well represented by the existing suite of claims, these behaviors can be implemented without changes to the Broker itself by simply extending the claim base class. Then, once developed, such a novel claim can easily be shared with all existing Movers.

#### Enforcement of the Contract

The Broker enforces the results of broking. During broking, the Broker replaces the usual conformation implementation with a *protected conformation*, which requires each Mover to supply credentials (*i*.*e*., its passport) to execute the modification. To supply its passport, the client creates an *unlock*, which is shared between the client and the conformation. When the Mover invokes any of the methods that require authentication, the conformation examines the active unlock to determine whether the change is allowed (Fig 4E).

Use of the unlock minimizes the overhead for an individual sampling move. An unlock is a small object that is allocated on the stack; as part of its construction, it receives a passport and protected conformation. As it is constructed, it pushes the passport onto a stack in the protected conformation. When a DoF change is made in the protected conformation, it checks only this top passport on the stack to see if the change is allowed. When the unlock goes out of scope (typically at the end of the client Mover’s “apply” function), the passport is popped from the stack.

In principle, if every developer of every client Mover were to follow the contract always, in every context, and without error, enforcement of the contract would be superfluous. It is unrealistic, however, to expect that developers will develop completely error-free Movers in a codebase that, at the time of this writing, includes nearly 1.8 million lines of C++ code maintained and developed by more than 100 active developers throughout North America, Europe, and Asia. Enforcing the contract creates a system that, much like type-safety and const-correctness in C++, creates a fail-fast feedback mechanism for catching mistakes in adherence to the contract quickly and at their source.

## Conclusions

In this paper, we introduce a new architecture that simplifies combining complex Movers with nontrivial needs from the fold tree into a single, concerted sampling strategy. We demonstrate our system’s flexibility and expressiveness by building several protocols that use a limited number of plug-and-play parts. Prior to this work, the design of such sophisticated protocols required significant time from an expert developer. By introducing an improved model of Mover interactions, where each Mover communicates with one central broking mechanism, we create an ecosystem where all Movers’ needs, independent of their combined complexity, can be represented simultaneously, if such a representation exists. Where no such representation exists, the system fails gracefully with an explanation of the failure, rather than silently producing meaningless results.

Simultaneous use of multiple constraint sets has historically proven useful[4,9,18,22,31,47], but has only been available to experienced Rosetta developers who must spend significant time developing a custom-made broking system for their application. We view the Broker as a twofold improvement: not only does it save experienced developers time, but it has the potential to democratize the development of sophisticated protocols, allowing scientists who are not experienced Rosetta C++ developers to experiment with simultaneous sampling procedures. We hope that the future of the Broker includes a flourishing community that develops ClientMovers and uses the Broker to solve interesting scientific problems while contributing to the continuing improvement of the platform as a whole.

As the Rosetta project grows in size and diversity, programmatic constraint compatibility—and thus the ability to rapidly prototype new sampling algorithms—becomes increasingly important. We view the Broker as an important step forward in the development of Rosetta as a powerful tool for macromolecular structure prediction—not just for expert developers, but for any researcher.

## Supporting Information

S1 File Scripts and flags that execute the protocols presented in this paper. This file contains each of the RosettaScripts XML and flags files required to run the protocols described in this paper. The protocol capture for this paper includes *all* input required for the protocol, including fragments and input coordinates for rigid chunks.
(DOCX)

## References

[1] Alder BJ, Wainwright TE. Studies in Molecular Dynamics. I. General Method. J Chem Phys. 1959;31: 459 10.1063/1.1730376

[2] Metropolis N, Rosenbluth AW, Rosenbluth MN, Teller AH, Teller E. Equation of State Calculations by Fast Computing Machines. J Chem Phys. 1953;21: 1087 10.1063/1.1699114

[3] Dimaio F, Echols N, Headd JJ, Terwilliger TC, Adams PD, Baker D. Improved low-resolution crystallographic refinement with Phenix and Rosetta. Nat Methods. 2013;10: 1102–1104. 10.1038/nmeth.2648 24076763 PMC4116791

[4] Zhang Z, Porter JR, Tripsianes K, Lange OF. Robust and highly accurate automatic NOESY assignment and structure determination with Rosetta. J Biomol NMR. 2014;59: 135–145. 10.1007/s10858-014-9832-4 24845473

[5] Kuhlman B, Dantas G, Ireton GC, Varani G, Stoddard BL, Baker D. Design of a novel globular protein fold with atomic-level accuracy. Science. American Association for the Advancement of Science; 2003;302: 1364–1368. 10.1126/science.1089427 14631033

[6] Correia BE, Loomis RJ, Carrico C, Jardine JG, Rupert P, Correnti C, et al Proof of principle for epitope-focused vaccine design. Nature. 2014;507: 201–206. 10.1038/nature12966 24499818 PMC4260937

[7] Röthlisberger D, Khersonsky O, Wollacott AM, Jiang L, DeChancie J, Betker J, et al Kemp elimination catalysts by computational enzyme design. Nature. Nature Publishing Group; 2008;453: 190–195. 10.1038/nature06879 18354394

[8] Roy A, Kucukural A, Zhang Y. I-TASSER: a unified platform for automated protein structure and function prediction. Nat Protoc. Nature Publishing Group; 2010;5: 725–738. 10.1038/nprot.2010.5 20360767 PMC2849174

[9] Song Y, Dimaio F, Wang RY-R, Kim DE, Miles C, Brunette T, et al High-resolution comparative modeling with RosettaCM. Structure. 2013;21: 1735–1742. 10.1016/j.str.2013.08.005 24035711 PMC3811137

[10] Azoitei ML, Correia BE, Ban Y-EA, Carrico C, Kalyuzhniy O, Chen L, et al Computation-guided backbone grafting of a discontinuous motif onto a protein scaffold. Science. American Association for the Advancement of Science; 2011;334: 373–376. 10.1126/science.1209368 22021856

[11] Siegel JB, Zanghellini A, Lovick HM, Kiss G, Lambert AR, St Clair JL, et al Computational design of an enzyme catalyst for a stereoselective bimolecular Diels-Alder reaction. Science. 2010;329: 309–313. 10.1126/science.1190239 20647463 PMC3241958

[12] Simons KT, Kooperberg C, Huang E, Baker D. Assembly of protein tertiary structures from fragments with similar local sequences using simulated annealing and bayesian scoring functions. J Mol Biol. 1997;268: 209–225. 10.1006/jmbi.1997.0959 9149153

[13] Vernon R, Shen Y, Baker D, Lange OF. Improved chemical shift based fragment selection for CS-Rosetta using Rosetta3 fragment picker. J Biomol NMR. Springer Netherlands; 2013;57: 117–127. 10.1007/s10858-013-9772-4 23975356

[14] Russel D, Lasker K, Webb B, Velázquez-Muriel J, Tjioe E, Schneidman-Duhovny D, et al Putting the pieces together: integrative modeling platform software for structure determination of macromolecular assemblies. PLoS Biol. 2012;10: e1001244 10.1371/journal.pbio.1001244 22272186 PMC3260315

[15] Berendsen HJ, van der Spoel D, van Drunen R. GROMACS: A message-passing parallel molecular dynamics implementation. Computer Physics Communications. 1995;91: 43–56. 10.1016/0010-4655(95)00042-E

[16] Adcock SA, McCammon JA. Molecular dynamics: survey of methods for simulating the activity of proteins. Chem Rev. 2006;106: 1589–1615. 10.1021/cr040426m 16683746 PMC2547409

[17] Dominguez C, Boelens R, Bonvin AMJJ. HADDOCK: A Protein−Protein Docking Approach Based on Biochemical or Biophysical Information. J Am Chem Soc. 2003;125: 1731–1737. 10.1021/ja026939x 12580598

[18] Lange OF, Baker D. Resolution‐adapted recombination of structural features significantly improves sampling in restraint‐guided structure calculation. Proteins. 2012 10.1002/prot.23245 PMC3310173 22423358

[19] Zanghellini A, Jiang L, Wollacott AM, Cheng G, Meiler J, Althoff EA, et al New algorithms and an in silico benchmark for computational enzyme design. Protein Sci. Cold Spring Harbor Laboratory Press; 2006;15: 2785–2794. 10.1110/ps.062353106 17132862 PMC2242439

[20] Miyamoto S, Kollman PA. Settle: An analytical version of the SHAKE and RATTLE algorithm for rigid water models. J Comput Chem. 1992;13: 952–962. 10.1002/jcc.540130805

[21] Hess B, Bekker H, Berendsen HJ, Fraaije JG, others. LINCS: a linear constraint solver for molecular simulations. J Comput Chem. John Wiley & Sons, Inc; 1997;18: 1463–1472. 10.1002/(sici)1096-987x(199709)18:12<1463::aid-jcc4>3.0.co;2-h

[22] Bradley P, Baker D. Improved beta-protein structure prediction by multilevel optimization of nonlocal strand pairings and local backbone conformation. Proteins. 2006;65: 922–929. 10.1002/prot.21133 17034045

[23] Leaver-Fay A, Tyka MD, Lewis SM, Lange OF, Thompson J, Jacak R, et al ROSETTA3: an object-oriented software suite for the simulation and design of macromolecules. Meth Enzymol. Elsevier; 2011;487: 545–574. 10.1016/B978-0-12-381270-4.00019-6 21187238 PMC4083816

[24] Mandell DJ, Coutsias EA, Kortemme T. Sub-angstrom accuracy in protein loop reconstruction by robotics-inspired conformational sampling. Nat Methods. 2009;6: 551–552. 10.1038/nmeth0809-551 19644455 PMC2847683

[25] Canutescu AA, Dunbrack RL Jr. Cyclic coordinate descent: A robotics algorithm for protein loop closure. Protein Sci. 2003;12: 963–972. 10.1110/ps.0242703 12717019 PMC2323867

[26] Gray JJ, Moughon S, Wang C, Schueler-Furman O, Kuhlman B, Rohl CA, et al Protein–Protein Docking with Simultaneous Optimization of Rigid-body Displacement and Side-chain Conformations. J Mol Biol. 2003;331: 281–299. 10.1016/S0022-2836(03)00670-3 12875852

[27] Meiler J, Baker D. ROSETTALIGAND: protein-small molecule docking with full side-chain flexibility. Proteins. 2006;65: 538–548. 10.1002/prot.21086 16972285

[28] André I, Bradley P, Wang C, Baker D. Prediction of the structure of symmetrical protein assemblies. Proc Natl Acad Sci USA. 2007;104: 17656–17661. 10.1073/pnas.0702626104 17978193 PMC2077069

[29] Azoitei ML, Ban YA, Kalyuzhny O, Guenaga J, Schroeter A, Porter JR, et al Computational design of protein antigens that interact with the CDR H3 loop of HIV broadly neutralizing antibody 2F5. Proteins. 2014;82: 2770–2782. 10.1002/prot.24641 25043744 PMC4437801

[30] Huang P-S, Ban Y-EA, Richter F, André I, Vernon R, Schief WR, et al RosettaRemodel: A Generalized Framework for Flexible Backbone Protein Design. Uversky VN, editor. J Mol Biol. 2011;6: e24109 10.1371/journal.pone.0024109 PMC3166072 21909381

[31] Sircar A, Gray JJ. SnugDock: paratope structural optimization during antibody-antigen docking compensates for errors in antibody homology models. PLoS Comput Biol. 2010;6: e1000644 10.1371/journal.pcbi.1000644 20098500 PMC2800046

[32] Lange OF, Rossi P, Sgourakis NG, Song Y, Lee H-W, Aramini JM, et al Determination of solution structures of proteins up to 40 kDa using CS-Rosetta with sparse NMR data from deuterated samples. Proc Natl Acad Sci USA. National Acad Sciences; 2012;109: 10873–10878. 10.1073/pnas.1203013109 22733734 PMC3390869

[33] Das R, André I, Shen Y, Wu Y, Lemak A, Bansal S, et al Simultaneous prediction of protein folding and docking at high resolution. Proc Natl Acad Sci USA. 2009;106: 18978–18983. 10.1073/pnas.0904407106 19864631 PMC2770007

[34] Fleishman SJ, Leaver-Fay A, Corn JE, Strauch E-M, Khare SD, Koga N, et al RosettaScripts: a scripting language interface to the Rosetta macromolecular modeling suite. PloS one. 2011;6: e20161 10.1371/journal.pone.0020161 21731610 PMC3123292

[35] Chaudhury S, Lyskov S, Gray JJ. PyRosetta: a script-based interface for implementing molecular modeling algorithms using Rosetta. Bioinformatics. 2010;26: 689–691. 10.1093/bioinformatics/btq007 20061306 PMC2828115

[36] Shen Y, Lange O, Delaglio F, Rossi P, Aramini JM, Liu G, et al Consistent blind protein structure generation from NMR chemical shift data. Proc Natl Acad Sci USA. 2008;105: 4685–4690. 10.1073/pnas.0800256105 18326625 PMC2290745

[37] Raman S, Lange OF, Rossi P, Tyka MD, Wang X, Aramini J, et al NMR structure determination for larger proteins using backbone-only data. Science. 2010;327: 1014–1018. 10.1126/science.1183649 20133520 PMC2909653

[38] Vijay-Kumar S, Bugg CE, Cook WJ. Structure of ubiquitin refined at 1.8 A resolution. J Mol Biol. 1987;194: 531–544. 3041007 10.1016/0022-2836(87)90679-6

[39] Ponting CP, Russell RR. THE NATURAL HISTORY OF PROTEIN DOMAINS. Annu Rev Biophys Biomol Struct. Annual Reviews 4139 El Camino Way, P.O. Box 10139, Palo Alto, CA 94303–0139, USA; 2003;31: 45–71. 10.1146/annurev.biophys.31.082901.134314 11988462

[40] Wollacott AM, Zanghellini A, Murphy P, Baker D. Prediction of structures of multidomain proteins from structures of the individual domains. Protein Sci. 2007;16: 165–175. 10.1110/ps.062270707 17189483 PMC2203296

[41] Berrondo M, Ostermeier M, Gray JJ. Structure prediction of domain insertion proteins from structures of individual domains. Structure. 2008;16: 513–527. 10.1016/j.str.2008.01.012 18400174 PMC2447813

[42] Moult J, Pedersen JT, Judson R, Fidelis K. A large-scale experiment to assess protein structure prediction methods. Proteins. 1995;23: ii–v. 10.1002/prot.340230303 8710822

[43] Inbar Y, Benyamini H, Nussinov R, Wolfson HJ. Combinatorial docking approach for structure prediction of large proteins and multi-molecular assemblies. Phys Biol. 2005;2: S156–65. 10.1088/1478-3975/2/4/S10 16280621

[44] Wang C, Bradley P, Baker D. Protein–Protein Docking with Backbone Flexibility. J Mol Biol. 2007;373: 503–519. 10.1016/j.jmb.2007.07.050 17825317

[45] Honegger A, Plueckthun A. Yet another numbering scheme for immunoglobulin variable domains: an automatic modeling and analysis tool. J Mol Biol. 2001 10.1006/jmbi.2001.4662 11397087

[46] Weitzner BD, Kuroda D, Marze N, Xu J, Gray JJ. Blind prediction performance of RosettaAntibody 3.0: Grafting, relaxation, kinematic loop modeling, and full CDR optimization. Proteins. 2014;82: 1611–1623. 10.1002/prot.24534 24519881 PMC4107143

[47] Azoitei ML, Ban Y-EA, Julien J-P, Bryson S, Schroeter A, Kalyuzhniy O, et al Computational design of high-affinity epitope scaffolds by backbone grafting of a linear epitope. J Mol Biol. 2012;415: 175–192. 10.1016/j.jmb.2011.10.003 22061265 PMC7105911

[48] Pettersen EF, Goddard TD, Huang CC, Couch GS, Greenblatt DM, Meng EC, et al UCSF Chimera—A visualization system for exploratory research and analysis. J Comput Chem. John Wiley & Sons, Inc; 2004;25: 1605–1612. 10.1002/jcc.20084 15264254

