# Supplementary material for: A Framework to Simplify Combined Sampling Strategies in Rosetta
Source: PLoS One. 2015 Sep 18;10(9):e0138220. doi: 10.1371/journal.pone.0138220 (PMC4575156; doi:10.1371/journal.pone.0138220)
Supplement: S1 File — This file contains each of the RosettaScripts XML and flags files required to run the protocols described in this paper. The protocol capture for this paper includes all input required for the protocol, including fragments and input coordinates for rigid chunks. (DOCX) [file pone.0138220.s001.docx]

In this supplement, we include scripts and flags used to execute the protocols given in the main text. They are all run using the rosetta_scripts executable included in Rosetta 3. We also direct the interested reader to the RosettaCommons wiki, where a programming guide and more detailed users’ manual can be found.

Furthermore, fully functioning examples of all of these protocols including recommended directory structures, input files, and fragment selections are included in the Rosetta demos repository, at the paths:

demos/protocol_capture/broker/{ubq,domain_insertion,snugdock}

## For *Ab initio* with Multiscale Constraints

The RosettaScripts XML that executes the protocol is as follows:

<ROSETTASCRIPTS>

<RESIDUE_SELECTORS>

<Index name="helix" resnums="17-40" />

</RESIDUE_SELECTORS>

<MOVERS>

<SwitchResidueTypeSetMover name="centroid" set="centroid" />

<FragmentJumpCM name="jumps" topol_file="1ubqA.top" />

<RigidChunkCM name="chunk" region_selector="helix" selector="helix"

template="1ubqA.pdb" apply_to_template="centroid" />

<AbscriptLoopCloserCM name="closer" fragments="frags/1d3z.frags3.dat.gz" />

<!-- Define the abinitio mover that will do torsion and beta-straind pairing insertions -->

<AbscriptMover name="abinitio" cycles=2 >

<!-- Abscript mover has special macros for defining the usual ab initio fragment movers, since there's three -->

<!-- and they're very stereotyped. You could use the <Stage> tag syntax to do it, but it's a pain. -->

<Fragments large_frags="frags/1d3z.frags9.dat.gz" small_frags="frags/1d3z.frags3.dat.gz" />

<!-- Add the mover "jumps" (fragment jumps) to stages I through IVb (i.e. all of them) -->

<Stage ids="I-IVb" >

<Mover name="jumps" />

</Stage>

</Abscriptmover>

<!-- Make the environment, and add the abinitio mover, followed by a loop closer. The loop closer obviously -->

<!-- Only gets run once at the end, so it's not inside the Abscript mover. -->

<Environment name="env" auto_cut=1 allow_pure_movers=1 >

<Register mover="chunk" />

<Apply mover="abinitio" />

<Apply mover="closer" />

</Environment>

<!-- It's good to quckly idealize structures that come out of broken-chain abinitio environment runs, -->

<!-- because sometimes the idealizer in the AbinitioLoopCloser doesn't quite get the loop closed. -->

<Idealize name="idealize" />

<!-- Switch to full atom and relax. Abrelax actually only uses 5 FastRelax repetitions, but the RosettaScripts default is 8-->

<SwitchResidueTypeSetMover name="fullatom" set="fa_standard" />

<FastRelax name="relax" repeats=5 />

</MOVERS>

<FILTERS>

</FILTERS>

<PROTOCOLS>

<Add mover="centroid" />

<Add mover="env" />

<Add mover="idealize" />

<Add mover="fullatom" />

<Add mover="relax" />

</PROTOCOLS>

</ROSETTASCRIPTS>

where the file 1ubqA.top contains the topology file specifying the native ubiquitin strand pairings, 1ubqA.pdb is the A chain of the structure with PDB code 1ubq, 1d3z.frag3.dat.gz and 1d3z.frag9.dat.gz are the fragments picked using the backbone chemical shifts used to produce the structure with PDB code 1d3z.

The protocol is executed with the following flags:

-parser:protocol fixed_core.xml

-in:file:fasta 1ubqA.fasta

-in:file:native 1ubqA.pdb

-out:path:pdb out_pdbs

where the above XML script is named fixed_core.xml, the sequence of ubiquitin is found in 1ubqA.fasta, and 1ubqA.pdb is the crystal structure of ubiquitin. Additional flags (such as those included in “denovo_flags” in the protocol capture) can also be included to promote the success of the modeling run, but are unnessecary for this proof of principle.

## Domain-Insertion Modeling

The RosettaScripts XML that executes the protocol is as follows:

<ROSETTASCRIPTS>

<RESIDUE_SELECTORS>

<Index name="host_domain_wo_linker" resnums="1-159,288-339" />

<Index name="host_domain_w_linker" resnums="1-159,295-346" />

<Not name="inserted_domain" selector="host_domain_w_linker" />

</RESIDUE_SELECTORS>

<MOVERS>

<SwitchResidueTypeSetMover name="centroid" set="centroid" />

<FragmentJumpCM name="jumps" topol_file="1uufA.top" />

<AbscriptMover name=abinitio cycles=2 >

<Fragments large_frags="1uufA.frag9" small_frags="1uufA.frag3" />

<Stage ids=I-IVb>

<Mover name=jumps/>

</Stage>

</AbscriptMover>

<RigidChunkCM name=chunk region_selector="host_domain_wo_linker" template="1uufA.pdb" selector="host_domain_w_linker" apply_to_template="centroid" />

<AbscriptLoopCloserCM name=closer fragments="1uufA.frag3" />

<Environment name=env auto_cut=1 >

<Register mover=chunk />

<Apply mover=abinitio />

<Apply mover=closer />

</Environment>

<SwitchResidueTypeSetMover name="fullatom" set="fa_standard" />

<FastRelax name="relax" repeats=5 />

</MOVERS>

<FILTERS>

</FILTERS>

<PROTOCOLS>

<Add mover=centroid />

<Add mover=env />

<Add mover=fullatom />

<Add mover=relax />

</PROTOCOLS>

</ROSETTASCRIPTS>

Where 1uufA.top contains the appropriate native beta strand topology, 1uufA.frag3 and 1uufA.frag9 contain the typical 3- and 9-mer fragments, and 1uufA.pdb contains the crystal structure of 1uufA. Note the use of residue selectors in the first section to handle the missing density in the crystal structure, which is present in the sequence used as input.

The flags used were as follows:

-parser:protocol domain_insertion.xml

-in:file:fasta 1uufA.fasta

-in:file:native 1uufA.pdb

-out:path:pdb out_pdbs

where domain_insertion.xml contains the script shown above, 1uufA.fasta contains the full sequence of 1uuf chain A (not that this differs from the residues modeled in 1uuf.pdb, which is missing some residues), and 1uufA.pdb contains chain A from the crystal structure with PDB code 1uuf.

## Flexible Backbone Multibody Docking

The RosettaScripts XML that executes the protocol is as follows:

<ROSETTASCRIPTS>

<RESIDUE_SELECTORS>

<Chain name="Ab_L" chains="L" />

<Chain name="Ab_H" chains="H" />

<Or name="Ab" selectors="Ab_L,Ab_H" />

<Chain name="Ag" chains="C" />

<Index name=CDRL_loops resnums="27L-39L,57L-77L,110L-136L" />

<Index name=CDRH_loops resnums="27H-39H,57H-77H,110H-136H" />

<And name=Ab_L_noloops selectors="Ab_L" >

<Not selector="CDRL_loops"/>

</And>

<And name=Ab_H_noloops selectors="Ab_H" >

<Not selector="CDRH_loops"/>

</And>

</RESIDUE_SELECTORS>

<MOVERS>

<!-- Define centers of mass for the antibody's mobile loops -->

<CoMTrackerCM name="Ab_L" mobile_selector="Ab_L" />

<CoMTrackerCM name="Ab_H" mobile_selector="Ab_H" />

<CoMTrackerCM name="Ag_com" mobile_selector="Ag" />

<!-- Define the movers that will be perturbing the rigid body DoFs between Ab chains and Ag -->

<UniformRigidBodyCM name="Dock_Ab_L"

mobile="Ab_L" stationary="star_center"

rotation_magnitude=2.5 translation_magnitude=0.05 />

<UniformRigidBodyCM name="Dock_Ab_H"

mobile="Ab_H" stationary="star_center"

rotation_magnitude=2.5 translation_magnitude=0.05 />

<UniformRigidBodyCM name="Dock_Ag"

mobile="Ag_com" stationary="star_center"

rotation_magnitude=5.0 translation_magnitude=0.05 />

<!-- Minimize all loops -->

<ScriptCM name=MinLoops >

<MinMover bb=1 chi=1 />

<!-- The RigidChunkCMs will prevent the MinMover from getting control, so we can just select the whole Ab -->

<TorsionClaim backbone=1 sidechain=1 control_strength=CAN_CONTROL selector="Ab" />

</ScriptCM>

<LoopCM name=closer style=refine algorithm="CCD" selector="CDRH_loops" />

<!-- Docking == 90% com recentering = 7% minimization = 2%, loop closure @ CDRH loops == 1% -->

<RandomMover name="dock_bag"

movers= "Dock_Ab_L,Dock_Ab_H,Dock_Ag,Ab_L,Ab_H,MinLoops,closer"

weights=".30, .30, .30, .045, .045, .02, .01" />

<GenericMonteCarlo name="dock" mover_name="dock_bag" scorefxn_name="talaris2013" temperature=2.0 trials=100 />

<Environment name=env auto_cut=1 >

<!-- We only register the antigen Center of Mass, because the antigen is fixed backbone -->

<!-- so we never need to update the CoM -->

<Register mover="Ag_com" />

<Apply mover="dock" />

<!-- These guy will close the loops we opened. -->

<Apply mover="closer" />

</Environment>

</MOVERS>

<FILTERS>

</FILTERS>

<PROTOCOLS>

<Add mover=env />

</PROTOCOLS>

</ROSETTASCRIPTS>

which requires no additional files. The flags used are as follows:

-parser:protocol snugdock.xml

-in:file:s 1ahw_AHO.pdb

-out:path:pdb out_pdbs

where snugdock.xml is the above XML script, 1ahw_AHO.pdb is the PDB 1ahw renumbered with the Aho number scheme and which the chains A and B renamed to H and L, respectively. Chain C is the antigen.
